# Multilevel En-Bloc Excision of Thoracic Spine Primary Chondrosarcoma Through an All-Posterior Approach: A Report of Two Cases

**DOI:** 10.7759/cureus.70884

**Published:** 2024-10-05

**Authors:** Rodrigo Muscogliati, Nigil Palliyil, Daniel Shou Chien Chin, Kedar Deogaonkar, Mohammad Daher, Elie Najjar, Nasir Quraishi

**Affiliations:** 1 Centre for Spinal Studies and Surgery, Queens Medical Centre, Nottingham University Hospitals NHS Trust, Nottingham, GBR; 2 Hull York Medical School, University of Hull, Hull-York Medical School, Hull, GBR

**Keywords:** chondrosarcoma spine, multilevel en-bloc spondylectomy, posterior only approach for spine en-bloc tumour excision, thoracic spine tumour, wide excision of spine tumours

## Abstract

Chondrosarcomas are malignant, cartilage-forming neoplasms. As these tumours are resistant to chemotherapy and radiotherapy, en-bloc excision of the tumour with wide margins is the only option that provides maximum disease-free survival and possible cure. We present two cases of primary chondrosarcoma of the thoracic spine treated by multilevel en-bloc excision through an all-posterior approach.

Case 1 describes a 48-year-old female who presented with mid-back pain for six months. MRI revealed an expansile lesion between T9 and T11; a biopsy confirmed this to be chondrosarcoma. Following a posterior-only approach, the entire tumour mass with the overlying pleura, part of the T9-T11 vertebral body and the posterior elements, as well as the posteromedial part of the ninth, tenth, and eleventh ribs, were removed en bloc.

Case 2 describes a 29-year-old male who presented with mid-back pain for five months. MRI revealed a lesion at T10, which was later confirmed on histopathological examination to be chondrosarcoma, affecting T9-T11. Using a posterior-only approach, the entire tumour mass, with part of T8-T11 was delivered en bloc.

Both patients made an uneventful recovery, and there were no signs of disease at 24 months post-operatively.

Despite being technically demanding, multilevel en-bloc tumour resection of the spine remains the mainstay in certain primary tumours, as it is potentially curative. Although the overall complication percentage following multilevel en-bloc resections was high, the local recurrence rate was significantly low. While most published articles recommend a posterior-only approach for limited disease, the cases in this report suggest that a posterior-only approach could also be viable for select cases of multilevel chondrosarcoma.

## Introduction

Chondrosarcomas are malignant, cartilage-forming bone neoplasms that account for 10% of all primary bone tumours [1]. Conventionally, chondrosarcomas are resistant to both chemotherapy and radiotherapy, making surgery the most optimal treatment [1]. Surgical excision of chondrosarcomas in the spine poses distinct surgical challenges due to its proximity to the spinal cord and major vascular structures [2], which are usually not present in peripheral sites. This necessitates meticulous surgical planning and technically demanding steps to achieve oncological control while preserving neurological function. The excision of chondrosarcomas utilizes en-bloc resections, which aim to surgically remove the tumour as an intact single piece, with a shell of healthy tissue, providing maximum disease-free survival [1].

Achieving en-bloc tumour excision in the spine poses challenges due to the spine's complex anatomy [2]. Previously, en-bloc spondylectomies have described advocating for a staged anterior-posterior approach [3]. However, due to the tumour's anatomical location and lack of anterior invasion, as well as the potential for reduced morbidity and better visualization of the spinal cord, a posterior-only approach was chosen for the two cases in this report. Additionally, Kawahara et al. found that the single-stage, all-posterior en-bloc spondylectomy was achievable for lesions located from T1 to L2 [4]. Nevertheless, multilevel en-bloc excision is more challenging and infrequently used due to the higher incidence of major perioperative complications [5]. In this study, we describe two cases of multilevel primary chondrosarcomas of the thoracic spine, treated with total en-bloc excision through an all-posterior approach.

## Case presentation

Case 1

**Figure 1:**
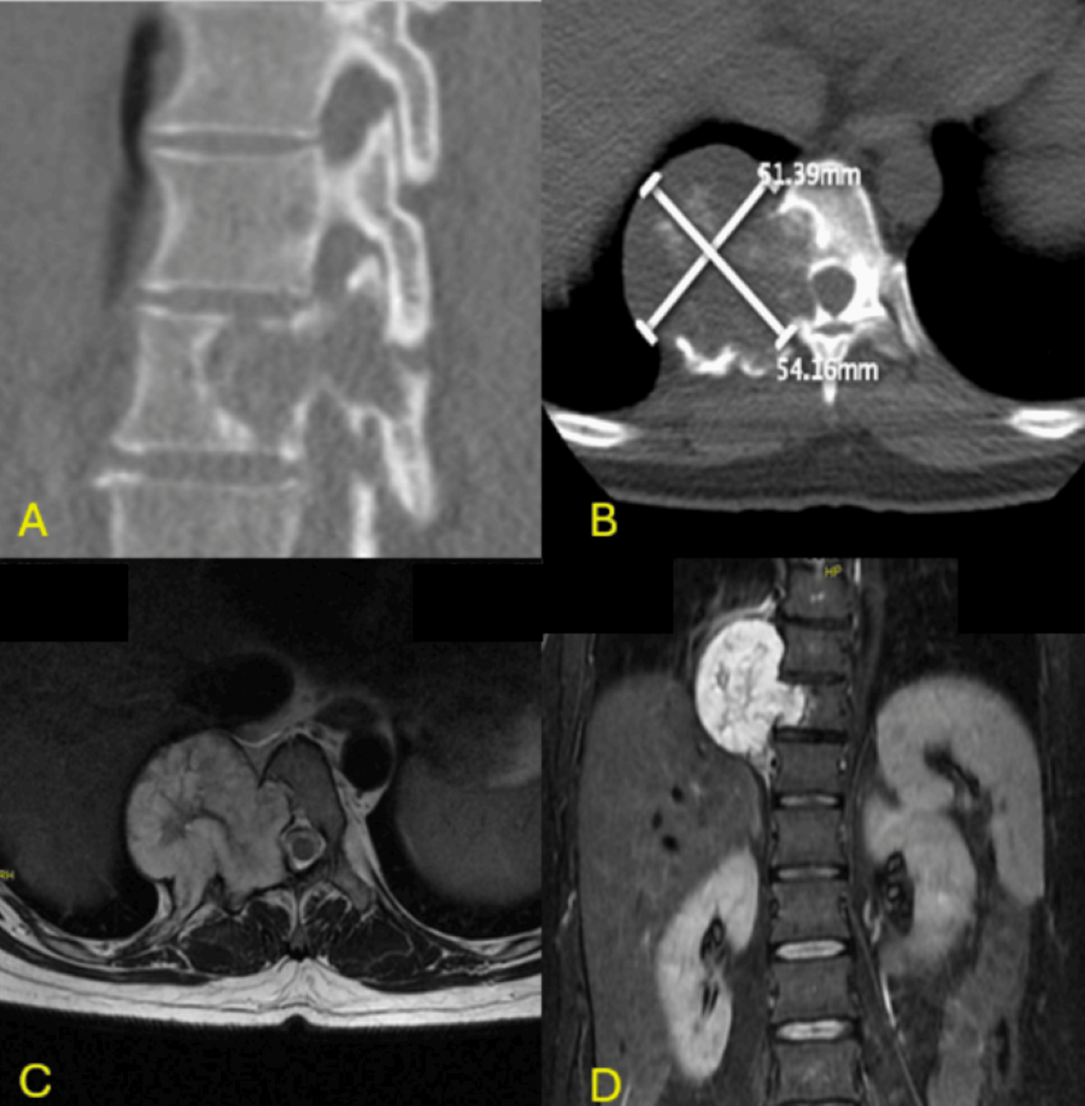
Radiographic images showing the chondrosarcoma pre-operatively 1A: CT sagittal view showing an osteolytic lesion involving the T10 vertebral body with extension to the posterior elements on the right side 1B: CT axial view showing an osteolytic lesion in the right half of the T10 vertebral body with extension to the pedicle and adjacent rib. 1C: MRI axial T2 image showing an expansive, lobulated lesion involving the right-sided posterior elements of the T10 vertebral body and the posterior aspect of the right tenth rib. The expansile lesion mildly effaces the right lateral margin of the spinal canal. 1D: MRI STIR coronal image showing an expansile lobulated lesion involving the right half of the T10 vertebral body. The lesion effaces the lateral aspect of the T9 and T11 bodies on the right side. STIR: short tau inversion recovery

A 48-year-old female patient presented with continuous pain in the mid-back region for six months. Physical examination revealed point tenderness over the thoracolumbar region. Her neurological examination was unremarkable. An MRI and CT scan revealed an expansile lesion involving the right half of the T10 vertebral body extending to both T9 and T11 (Figures 1A-1D). A CT-guided biopsy and histopathological examination of the lesion revealed the diagnosis of chondrosarcoma. A positron emission tomography-computed tomography (PET-CT) scan revealed this to be a solitary lesion. Pre-operative angiography with embolization was performed 24 hours before surgery to delineate tumour vasculature and occlude the feeder vessels.

Surgical technique

**Figure 2:**
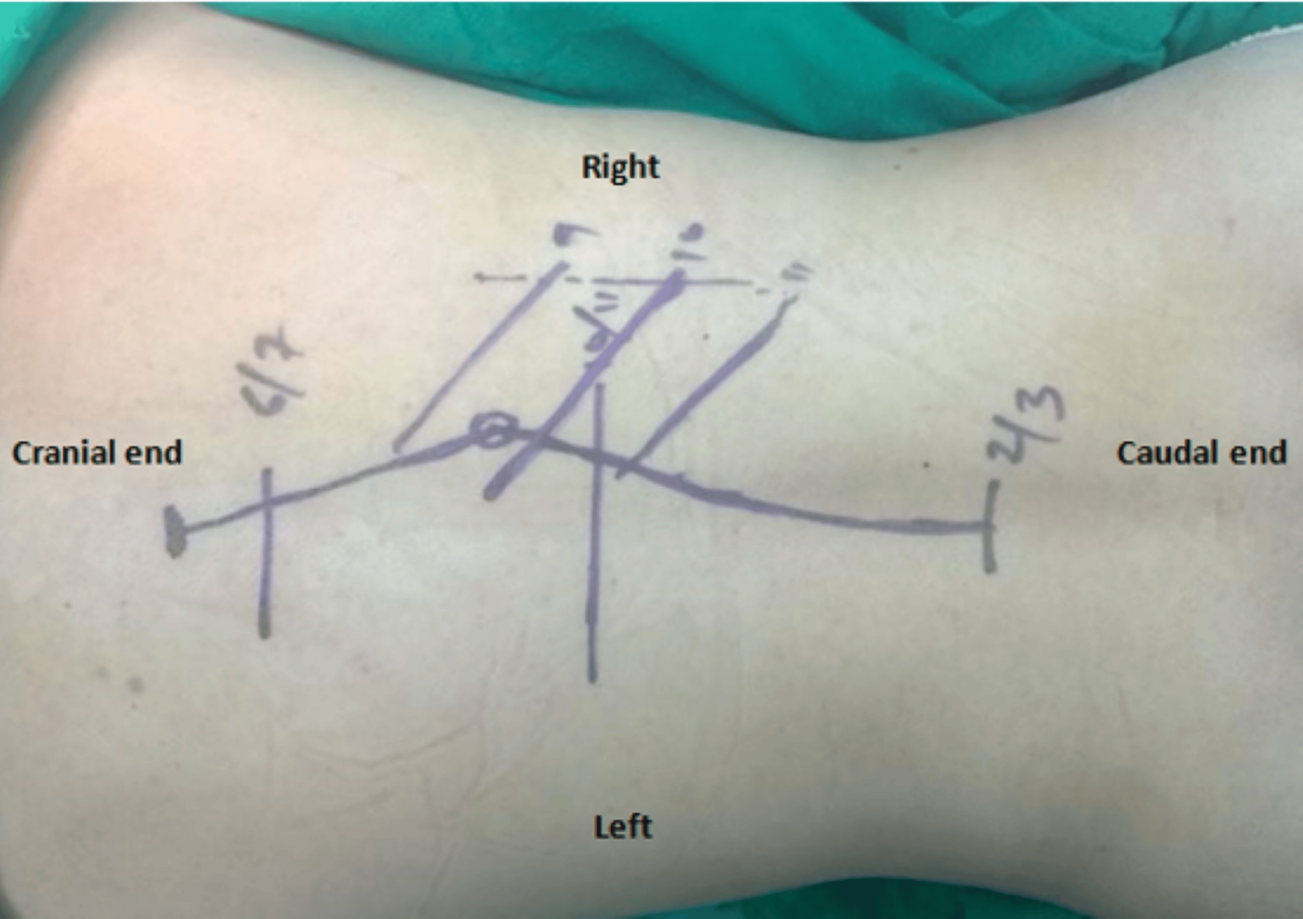
Skin markings pre-surgically Preoperative skin marking showing the curvilinear incision incorporating the biopsy track

The patient was placed in a prone position over bolsters, with her head supported in a Mayfield’s clamp in a slightly reverse Trendelenburg position. A curvilinear incision was marked on the skin, to include the biopsy track (Figure 2). Subperiosteal dissection was carried out on the unaffected left side to expose the T6-L2 lamina. On the right side, T6-L2 laminae were exposed, leaving behind a sleeve of soft tissue behind the T10 lamina to avoid a tumour breach. The biopsy track was excised along with a margin of skin and soft tissue.

**Figure 3:**
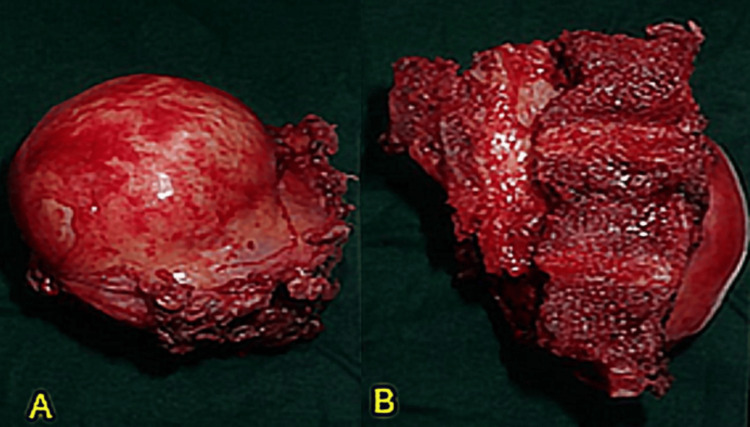
Gross intact specimen Gross intact specimen of chondrosarcoma after being excised 3A: Lateral sagittal view of the tumour 3B: Medical sagittal view of the tumour

Pedicle screws were inserted bilaterally using fluoroscopic guidance at the T6-T8 and T12-L2 pedicles. A rod was inserted on the left side. Then, T9-T11 hemilaminectomies were performed in a piecemeal fashion on the uninvolved left side to expose the dura and nerve roots, ligating the latter on the left side. On the right side, the ninth, tenth, and eleventh ribs were exposed posteriorly and cut approximately 4 cm from the costo-transverse joint. The T8-T9 and T11-T12 disc spaces were prepared using end-plate curettes, excising their anterior and posterior longitudinal ligaments. Oblique osteotomy cuts were made through the vertebral bodies of T9-T11 from the left side to remove the tumour mass with an adequate margin. The segmental vessels were ligated as identified; T9-T11 nerve roots were ligated and divided on the right side. The entire tumour mass with the overlying pleura, part of the T9-T11 vertebral body as well as the posteromedial part of the ninth, tenth, and eleventh ribs were removed en-bloc (Figures 3A-3B). Anterior reconstruction was performed with the placement of a titanium mesh cage across the defect. Prolene mesh was used to cover the defect in the pleura after placing the chest tube drain in the second rod, a cross-link was inserted, and the wound was closed in layers.

**Figure 4:**
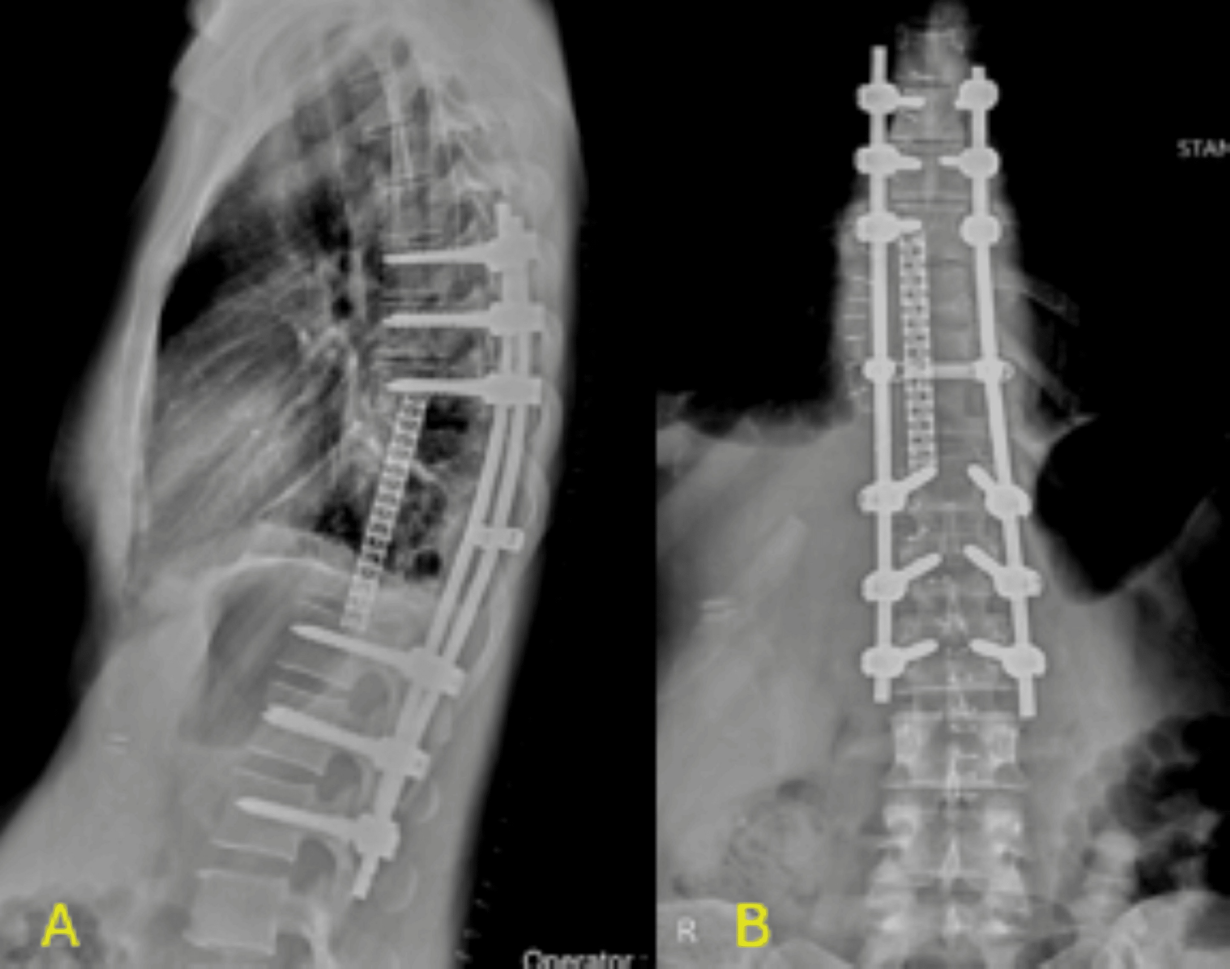
Post-operative X-rays Immediate post-operative x-rays showing the mesh cage anteriorly and posterior instrumentation 4A: Sagittal X-rays showing the mesh cage anteriorly and posterior instrumentation 4B: Coronal X-rays showing the mesh cage and spinal instrumentation

**Figure 5:**
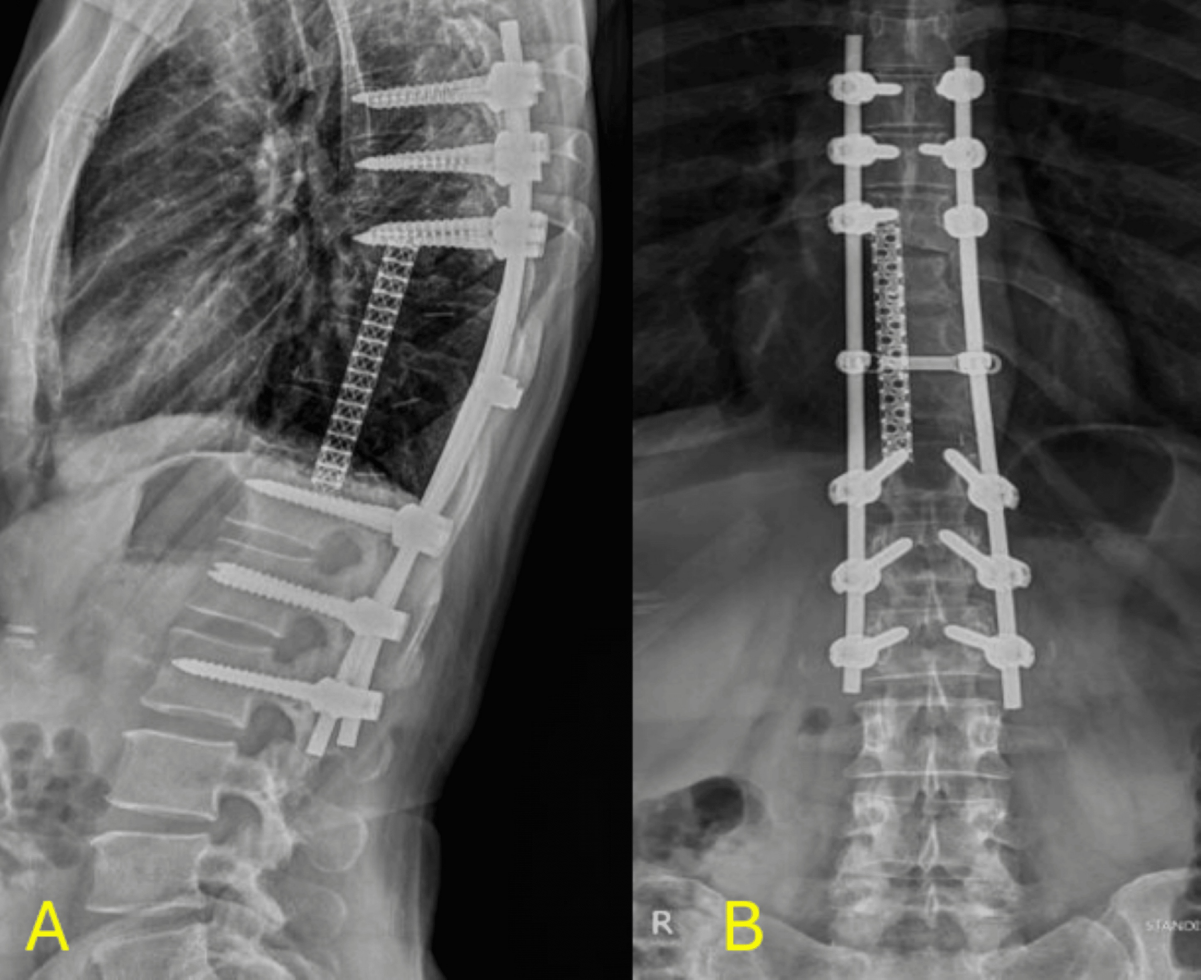
X-rays at the one-year follow-up X-ray at the one-year follow-up showing the integrity of the mesh cage's anterior and posterior instrumentation 5A: Sagittal view X-ray of the mesh and spinal instrumentation at the one-year follow-up 5B: Coronal view X-ray of the mesh and spinal instrumentation at the one-year follow-up

The patient was kept in the ICU for 48 hours. Her post-operative course was uneventful, and the patient was discharged home 12 days after her operation, with their immediate post-operative X-ray being shown in (Figures 4A-4B). At 24 months post-operatively, there was no sign of recurrence or complication, with X-rays taken at one year post-operatively showing stable construct with no implant failures (Figures 5A-5B). Chondrosarcomas of the thoracic spine have a reported recurrence rate of 32-58% following en-bloc resection [6]. Regular follow-up imaging, including MRI and CT, was conducted at six-month intervals, contributing to the early detection of any recurrence. Given the absence of recurrence at 24 months, these strategies appear to be effective, though longer follow-up is essential due to the long-term nature of chondrosarcoma recurrence.

Case 2

**Figure 6:**
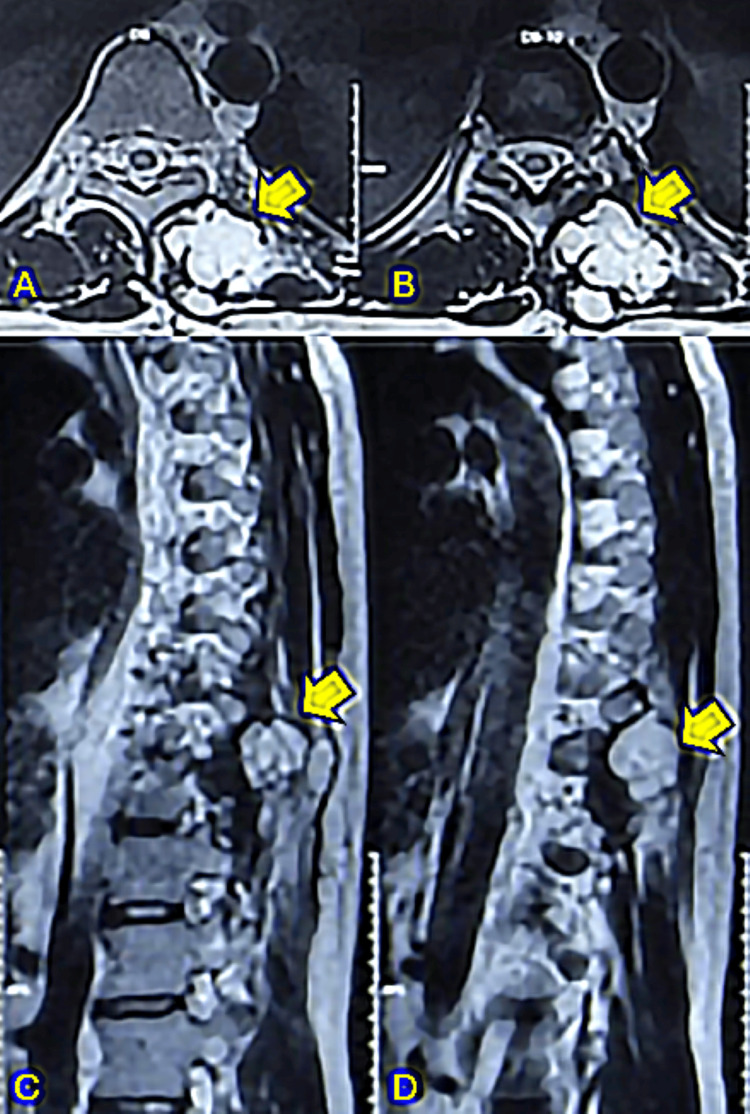
T2-weighted MRI showing the chondrosarcoma at levels T9, T10, and T11 6A and 6B: T2-weighted MRI axial cuts showing a well-defined, lobulated, hyperintense lesion predominantly involving the left paraspinal muscles at the T9, T10 vertebral body level with associated involvement of the T9, T10, T11 spinous processes, left transverse processes and pedicles of the T9, T10 vertebrae, and posteromedial aspects of the respective left ribs. 6C and 6D: MRI T2-weighted sagittal cuts showing a well-defined, lobulated, hyperintense lesion involving the posterior elements and paraspinal muscle at T9, T10, and T11.

**Figure 7:**
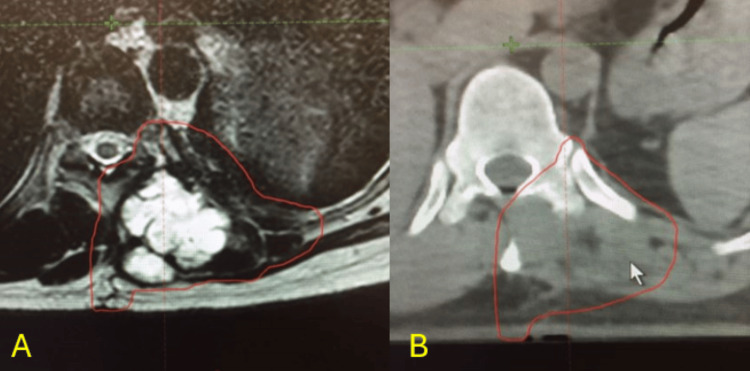
Pre-operative planning to mark the boundaries of excision Radiographic imaging used during surgical planning to mark the boundaries to perform the en-bloc excision of the tumour 7A: Tumour delineation under MRI 7B: Tumour delineation under CT scan

This is the case of a 29-year-old male patient who presented with a dull mid-back ache for five months. His MRI revealed a lesion in the T10 vertebral posterior elements for which a needle biopsy was performed, which reported it as an enchondroma. The patient underwent posterior T10 decompression surgery elsewhere. However, the histopathological examination of the tissue obtained from surgery revealed chondrosarcoma. Four months after the surgery, the patient presented to the clinic; the physical examination revealed diffuse tenderness over the thoracolumbar spine. His neurological examination was normal; a repeat MRI revealed a well-defined lesion predominantly involving the left paraspinal muscles at the T9, T10 vertebral body level with an associated involvement of the T9-T11 spinous process, left transverse processes and pedicles of the T9, T10 vertebrae as well as the posteromedial aspects of respective left ribs (Figures 6A-6B). A CT scan was done for detailed surgical planning (Figures 7A-7B). An angiographic embolization was also performed 24 hours before surgery.

Surgical technique

**Figure 8:**
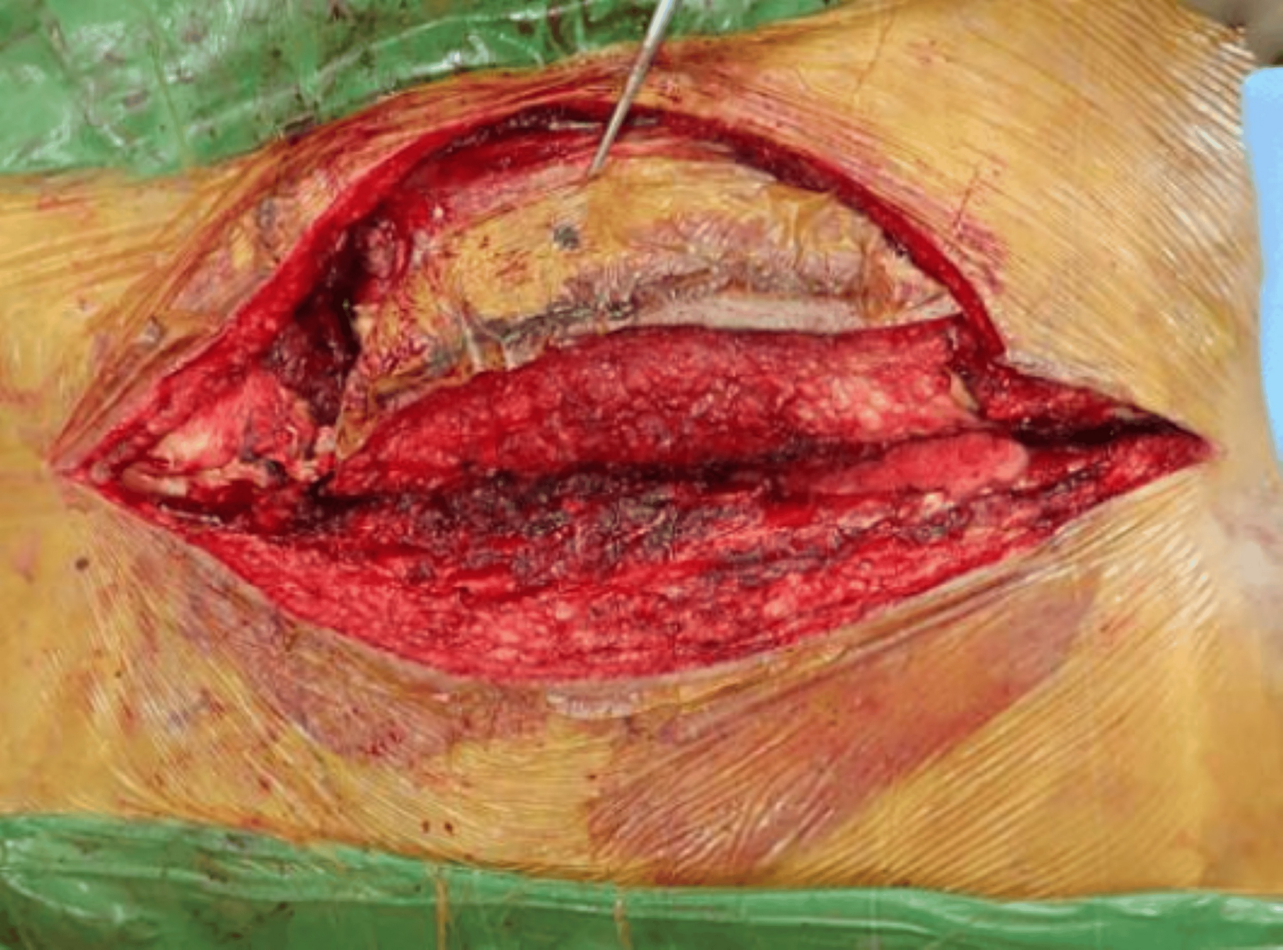
The surgical incision with wide margins Wide margins were taken on the left side to incorporate the previous surgical scar

The patient was positioned similarly to the previous case. A midline incision ellipting the previous scar with a 1 cm margin on the right and a 3 cm margin on the left was utilised (Figure 8). The right-sided paraspinal muscles were elevated away from the spine and a piecemeal laminectomy was performed on the unaffected right side at T8-T12 to expose the dura and nerve roots. Pedicle screws were inserted from T6 to L2 with a right-sided rod using fluoroscopic guidance. On the left side, the chest wall muscles were cut longitudinally 3 cm from the palpable tumour margin. The eighth to twelfth ribs were exposed and cut 4 cm from the costovertebral junction to gain better access to the thoracic cavity. The superior facet at the T10 level and the T12 inferior facet on the left were transected and the thoracic cavity entered, staying close to the inner side of the chest wall. The T10-12 nerve roots were transacted and intercostal vessels were ligated.

**Figure 9:**
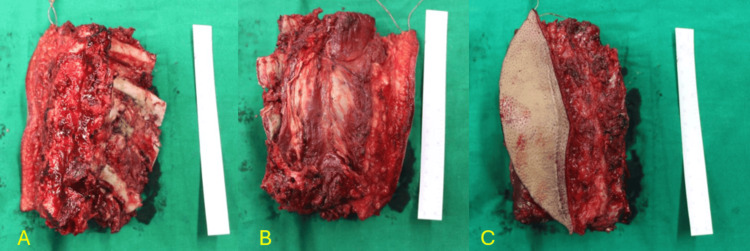
Gross intact specimen after the en-bloc removal of the tumour 9A: Sagittal view of the gross intact specimen after removal en-bloc 9B: Anterior coronal view of the gross intact specimen after removal en-bloc 9C: Posterior coronal view of the gross intact specimen after removal en-bloc

Using an osteotome at the T11-T12 pedicles, the specimen was excised (Figures 9A-9C). The pleura was directly repaired. The left rod and cross-links were positioned.

**Figure 10:**
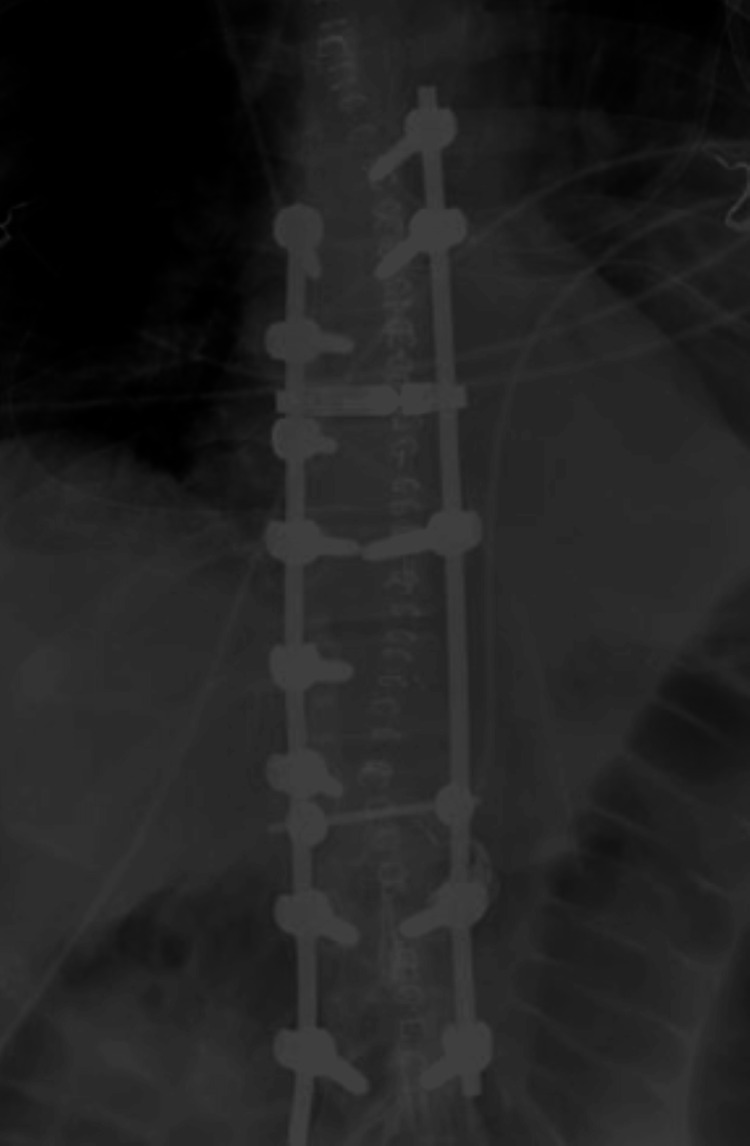
Post-operative X-ray Immediate post-operative coronal X-ray showing posterior instrumentation

**Figure 11:**
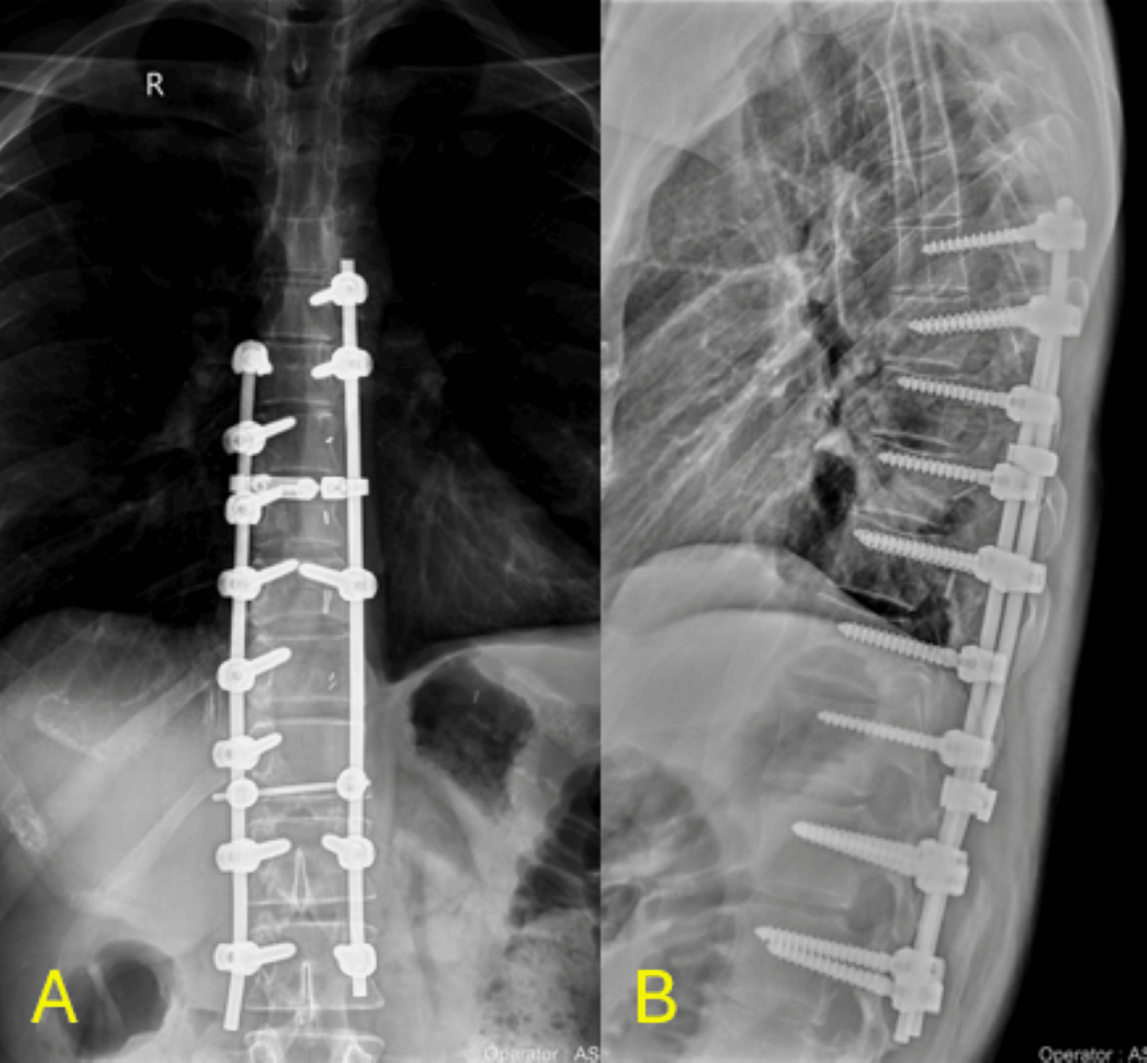
Follow-up X-ray eight months after surgery At the upper end of the construct, unilateral instrumentation was chosen to avoid creating a long lever arm between two screws, which could compromise the stability of the construct. By adding one additional screw distally, we ensured greater biomechanical stability and reduced the risk of hardware failure. 11A: Coronal X-ray showing spinal instrumentation at eight months post-surgically 11B: Sagittal X-ray showing spinal instrumentation at eighth months post-surgically

His post-operative period was uneventful, with his immediate post-operative X-ray shown in Figure 10. He was kept in the ICU for 48 hours, and he was discharged 10 days following his procedure. Twenty-four months post-operatively, there were no recurrence or complications, with Figures 11A-11B showing his X-rays eight months post-operatively.

## Discussion

When comparing both cases, the initial recurrence in the second case after prior surgery emphasizes the importance of achieving negative margins during initial surgery, as incomplete excision or tumour spillage can lead to recurrence. Both cases had similar tumour grades, but the recurrence may have been influenced by tumour location and the surgical approach in the initial procedure [7].

As described by Liljenqvist et al. in 2008, en-bloc surgeries of the spine were popularized in 1994 by Tomita et al. and Fidler [8-10]. Tomita et al. explored a posterior-only approach to reach the spine while Filder opted to use a mixed anterior-posterior approach [8-10]. The main advantage of the anterior-posterior approach is that it allows better visualization of the spinal anterior elements allowing safer removal of these structures [11,12]. Furthermore, discectomies, resection of the anterior longitudinal ligament and anterior column reconstruction are easier under an anterior approach [10].

On the other hand, the posterior-only approach allows tumour excision and circumferential reconstruction in a single stage, reducing operative time and post-operative morbidity [2]. Furthermore, the spinal cord is in direct vision throughout the surgery, especially during anterior spinal column osteotomy, corpectomy and spinal reconstruction [3]. However, the main disadvantage of a posterior-only approach is that it relies on the tumour’s anatomy, with too much tumour anterior extension making this approach unlikely to achieve safe margins [13].

There are, however, major surgical differences when comparing a posterior-only to an anterior-posterior approach. As shown by Boriani et al., 10.6% of patients who underwent a posterior-only approach suffered from surgery-related complications compared to 48.3% who had complications from an anterior-posterior approach, with 11.5% of the complications being directly caused by an anterior exposure [14,15]. For instance, in an anterior thoracotomy with spine exposure, there is a mortality rate of 3.2%. In addition, there is a 1-5% risk of cord ischemia with paraparesis or paraplegia in operations where there is bilateral disruption of segmental vessels when exposing the spine [16,17]. Furthermore, atelectasis, pneumonia and pneumothorax may be present in up to 50% of patients post-surgery [18]. In retroperitoneal and transperitoneal approaches, damage to the iliac vessels and inferior vena cava can occur at a rate of 2-4% [19].

In fact, Luzzati et al. report high rates of complications when performing the anterior-posterior approach to en-bloc, with a complication rate of 78%, of which 71% recovered [12]. On the other hand, Wang et al. showed a 75% complication rate after a posterior-only approach, with pleural defects being the main complication (62.5) [20]. In the cases presented in this report, pleural defects were avoided due to careful handling of the pleura during dissection, ensured by careful exposure and minimizing traction on the tissue. Additionally, we utilized a subperiosteal dissection technique, which allowed us to mobilize the pleura away from the surgical field without causing damage. While Wang et al. do not report on the resolution of these complications, they show that 69% of the patients were alive with no mention of disease recurrence at a mean follow-up of 34 months [20].

## Conclusions

Overall, there is limited evidence comparing the posterior-only approach to the anterior-posterior approach to en-bloc, even though the latter has well-recognized morbidities. While most published articles recommend a posterior-only approach for limited disease, showing lower complication rates when compared to a mixed approach, our cases in this report suggest that a posterior-only approach could also be viable for select cases of multilevel chondrosarcomas. However, it is important to note that a successful posterior-only approach to the en-bloc removal of chondrosarcomas in the spine relies on tumour anatomical factors, such as anterior invasion, as well as surgical expertise. Lastly, it is important to follow up on these patients with appropriate imaging due to the high recurrence rates of chondrosarcomas.
